# Association of Interleukin-8 with Cachexia from Patients with Low-Third Gastric Cancer

**DOI:** 10.1155/2009/212345

**Published:** 2009-11-23

**Authors:** Bo Song, Dianliang Zhang, Shuchun Wang, Hongmei Zheng, Xinxiang Wang

**Affiliations:** ^1^Department of General Surgery, Affiliated Hospital of Qingdao University Medical College, Qingdao 266003, China; ^2^Yantai Yantaishan Hospital, Yantai 264000, China; ^3^Yantai Chefoo Area Directly Subordinate Organ Hospital, Yantai 264000, China

## Abstract

*Background*. Interleukin (IL)-8 has been implicated in the development of cancer cachexia. The polymorphism of IL-8 gene, which may affect the production level of IL-8, may be associated with cancer cachexia. *Methods*. The serum IL-8 level in our study was examined by radioimmunoassay. We also analyzed single nucleotide polymorphisms (SNPs) −251 A/T and +781 C/T of IL-8 gene, using polymerase chain reaction-restriction fragment length polymorphism (PCR-RFLP). *Results*. The serum levels of IL-8 were significantly elevated in patients with low-third gastric cancer compared with controls, and were further up-regulated in patients with cachexia than those without (*Z* = −3.134, *P* = .002). A significantly increased frequency of +781 T allele was noted in patients with cachexia (OR = 2.247, 95% CI: 1.351–3.737, *P* = .002). The +781 TT genotype was observed to be associated with a significantly increased risk of cachexia (OR = 3.167, 95% CI: 1.265–7.929, *P* = .011), and with odds ratio of 3.033 (95% CI: 1.065–8.639, *P* = .038) for cachexia after adjusting for potential confounding factors. Meanwhile, haplotype analysis indicated a borderline positive association between T^251^T^781^ haplotype and cachexia as compared with the T^251^C^781^ haplotype (OR = 4.92, 95% CI: 1.00–24.28;, *P* = .053). *Conclusions*. IL-8 appears to be associated with cachexia from patients with low-third gastric cancer.

## 1. Introduction

Cachexia is a complex metabolic syndrome associated with many diseases such as cancer, acquired immunodeficiency syndrome (AIDS), parasitic diseases, autoimmune disorders, chronic heart failure, or sepsis [1, 2]. Patients with cachexia are characterized by anorexia, early satiety, severe weight loss, weakness, anemia, and edema [1, 3]. The most devastating effect of cachexia is a progressive loss of body weight, resulting in severe depletion of both adipose tissue and skeletal muscle [4]. But unlike the situation in starvation, nutritional supplementation has little or no impact on cachexia [5]. Cancer cachexia—which has a negative impact on patient's quality of life, therapy and survival—is the most common reason of cancer-related deaths. It is estimated that 20% of patients with malignant diseases die from the effects of cachexia rather than tumor burden [6, 7].

The mechanism of cancer cachexia remains not well know. In humans, there is an increasing evidence that cytokines, including tumor necrosis factor alpha (TNF-*α*), interleukin (IL)-1, IL-6, IL-8 and interferon gamma (IFN-*γ*) may participate in the cause or the development of cancer cachexia [8–13].

IL-8, a member of the CXC chemokine family, was originally identified as a potent chemoattractant for neutrophils and lymphocytes [14, 15]. Subsequent studies confirmed that IL-8 could also induce angiogenesis, tumor growth, invasion, and potential metastasis in cancer [16]. Recently, the associations between increased serum IL-8 level and cachexia have been demonstrated in several cancers [17, 18]. Some studies have reported that the two common polymorphisms of IL-8 gene, −251 A/T, and +781 C/T are associated with altered transcription levels of IL-8 [19–22], and the polymorphisms of IL-8 gene have been implicated in the susceptibility to a range of cancers [23–25].

It has been confirmed that the IL-6 gene polymorphisms play an important role in the development of cancer cachexia [26, 27], and the polymorphism of IL-1 *β* is associated with the cachexia from locally advanced gastric cancer [28]. However, no study has examined the association between genetic polymorphisms in IL-8 gene and cachexia. Working from the assumption of substantial inflammatory contribution to cancer cachexia, we evaluated the effects of serum IL-8 level and the two common −251 A/T and +781 C/T polymorphisms of the IL-8 gene on the susceptibility of cachexia from patients with low-third gastric cancer.

## 2. Materials and Methods

### 2.1. Patients

All of the consecutive patients with low-third gastric cancer admitted to our hospital between April 1, 2008 and October 1, 2008 were prospectively considered. All cases were Chinese Han Population, and were verified as low-third gastric adenocarcinoma by histopathological examination. The exclusion criteria were the following: (1) age >75 years, (2) anorexia nervosa, (3) pyloric obstruction (4) surgery, radiotherapy, or chemotherapy during the previous 4 weeks, (5) other active medical conditions (major gastrointestinal disease, chronic heart failure, hepatic failure and renal failure, uncontrolled diabetes, infections, and HIV).

Patients were investigated for the presence of smoking habits, drinking water status, weight loss, food intake, and C-reactive protein (CRP) levels. We defined “current” as persons who were current smokers, “ex-” as those who had smoked in the past, and “never” as those who had never smoked at anytime in their lives. Cancer stages were classified according to the guideline of International Union Against Cancer (UICC) TNM system [29].

All patients were divided into two groups cachectic patients and noncachectic patients. Patients were considered cachectic if they had met all 3 factors of the multifactor cachexia profile [30]: (1) weight loss (≥10% of their preillness stable weight within 6 months), (2) low food intake (≤1500 kcal/d), (3) systemic inflammation (CRP ≥10 mg/L).

The controls were recruited from our hospital attendees with no family history of gastric cancer and without any malignant diseases and infectious disorders. Informed consent was obtained from all participating subjects, and the Medical Ethics Committee of Affiliated Hospital of Qindao University approved the study.

### 2.2. Assessment of H Pylori Status

^14^C-UBT test was repeated two times for enrolled patients and controls by a single team of specialized staff. None of the enrolled subjects had ever been treated for H pylori eradication.

### 2.3. Cytokine Detection

Peripheral venous blood samples were collected at 6 a.m. on the first day after admission and all participants were in a fasting state. The levels of the cytokines were measured by a radioimmunoassay using Interleukin Radioimmunoassay Kit (North Biotechnology Research Institute, Beijing, China) according to the manufacturer's instructions.

### 2.4. DNA Extraction

Genomic DNA was extracted from EDTA-anticoagulated peripheral blood leukocytes using Wizard Genomic DNA Purification kit (Promega) according to the manufacturer's instructions.

### 2.5. Determination of IL-8 Genotype

The IL-8 −251 A/T and +781 C/T genotypes were determined by using a polymerase chain reaction-restriction fragment length polymorphism method (PCR-RFLP), and the PCR primers were designed as described previously.

### 2.6. IL-8 −251 A/T

**Figure 1:**
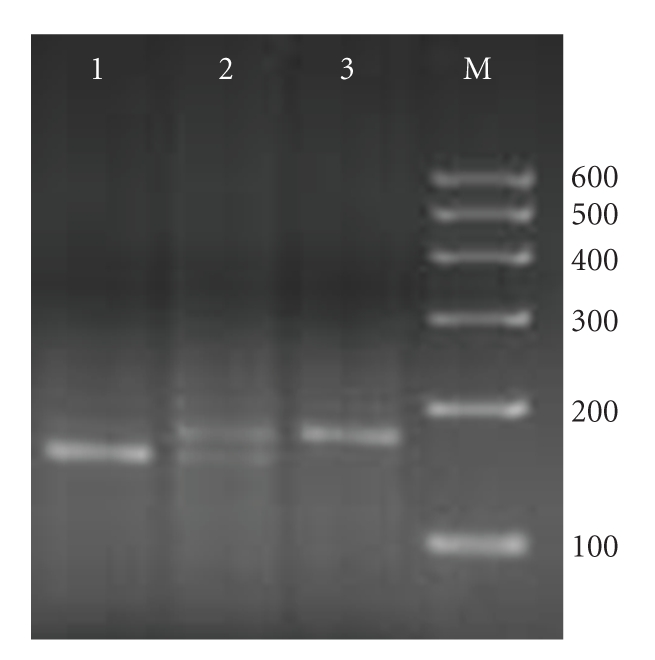
PCR-RFLP assay for analyzing the −251 A/T polymorphism of IL-8. Lanes 1 and 6 of heterozygous AT genotype showed three fragments of 173, 152, and 21 (not shown) bp; lane 4 and 7 homozygous of TT genotype showed only one fragment of 173 bp; lanes 2, 3 and 5 of homozygous AA genotype showed two fragments of 152 and 21 (not shown) bp. Lane M was loaded with 100–600 bp DNA markers.

Forward, CCATCATGATAGCATCTGT; reverse, CCACAATTTGGTGAATTATTAA [31] (Shanghai Sangon Biological Engineering Technology & Services Co.). PCR conditions: 94°C 5 minutes; 35 cycles of 30 seconds at 94°C, 55 seconds at 57°C, 1 minute at 72°C; 72°C 8 minutes. PCR products were digested with AseI (MBI Fermentas) for 4 hours at 37°C, producing fragments of 152 and 21 bp for allele A, or 173 bp for allele T; the fragments were visualized by electrophoresis on a 3% agarose gel stained with 0.1% ethidium bromide (Figure 1).

### 2.7. IL-8 +781 C/T

**Figure 2:**
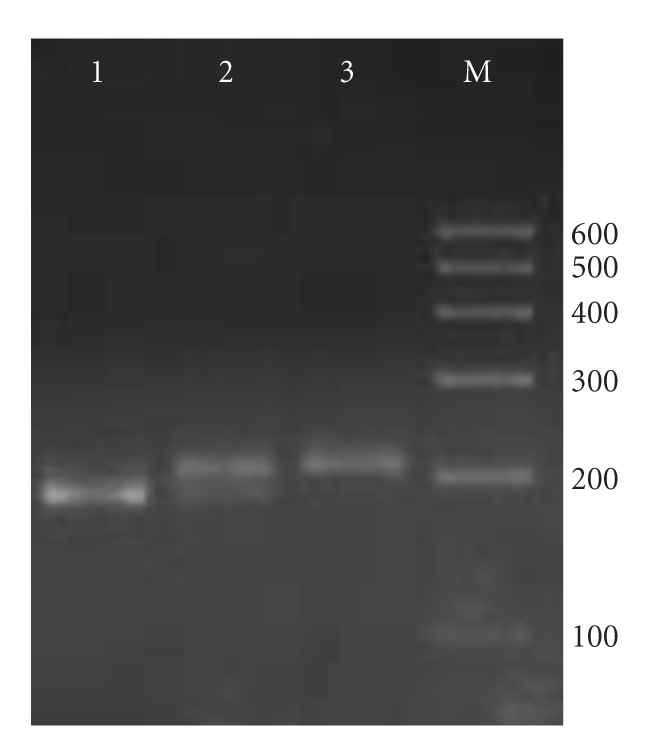
PCR-RFLP assay for analyzing the +781 C/T polymorphism of IL-8. Lanes 4 and 5 of heterozygous CT genotype showed three fragments of 203, 184, and 19 (not shown) bp; lane 1 and 3 of homozygous TT genotype showed only one fragment of 203 bp; lanes 2 and 6 of homozygous CC genotype showed two fragments of 184 and 19 (not shown) bp. Lane M was loaded with 100–600 bp DNA markers.

Forward, CTCTAACTCTTTATATAGGAATT; reverse, GATTGATTTTAT CAACAGGCA [31] (Shanghai Sangon Biological Engineering Technology & Services Co.). PCR conditions: 94° 8 minutes; 35 cycles of 30 seconds at 94°C, 30 seconds at 52°C, 1 minute at 72°C; 72°C 10 minutes. PCR products were digested with EcoRI (MBI Fermentas) for 4 hours at 37°C, producing fragments of 184 and 19 bp for allele C, or 203 bp for allele T, the fragments were visualized by electrophoresis on a 3% agarose gel stained with 0.1% ethidium bromide (Figure 2).

### 2.8. Statistical Analysis

The serum IL-8 levels were expressed as Mean ± SE and compared with Mann-Whitney U test. Demographic and clinical data between groups were compared by 2-sample *t* test and by *χ* ^2^** test. Genotype distributions for each polymorphism were first compared to values predicted by the Hardy-Weinberg equilibrium through *χ* ^2^ test. Genotype and allele frequencies among groups were compared using the *χ* ^2^ test and Fisher's exact test when appropriate, and odds ratios (OR) and 95% confidence intervals (CIs) were calculated to estimate the relative risk conferred by a particular allele and genotype. Haplotypes and linkage disequilibrium (LD) were determined based on the expectation-maximization algorithm using the SNPStats program (available at http://bioinfo.iconcologia.net/SNPstats/ provided in the public domain by the Biostatistics and Bioinformatics Unit, Catalan Institute of Oncology, Barcelona, Spain). A model of multiple logistic regression with variable entry criterion: *P* > .05 and variable removal criterion: *P* < .1 was also applied for identification of independent predictors of the cachexia.

*P* values were two sided and *P* < .05 was considered significant. The SPSS statistical software package version 12.0 was used for all of the statistical analyses.

## 3. Results

### 3.1. Characteristics of the Study Population

On the basis of the selection criteria, 125 patients with low-third gastric cancer were studied (61 cachectic patients). The characteristics of the study patients are shown in Table 1. Carcinoma stage was noted to be significantly different between the two groups (*P* < .001).

The clinical characteristics of the controls and the patients are shown in Table 2. No significant association was found between the frequency of smoking status, poor drinking water status and Helicobacter pylori infection. The two groups were matched for age and sex.

### 3.2. Serum IL-8 level in Patients and Controls

**Figure 3:**
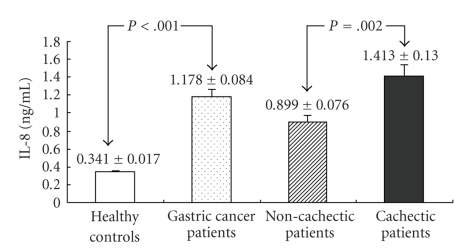
Serum levels of cytokines in gastric cancer patients and healthy subjects.

The serum level of IL-8 is presented in Figure 3. The IL-8 level in patients with low-third gastric cancer was significantly higher than that in the healthy controls (*Z* = −8.186, *P* < .001), and was further up-regulated significantly in cachectic patients compared with non-cachectic patients (1.413 ± 0.130 ng/mL versus 0.899 ± 0.076 ng/mL, *Z* = −3.134, *P* = .002). In a logistic regression analysis adjusted for actual weight and carcinoma stage, IL-8 was associated with odds ratio of 4.480 (95% CI: 1.527–13.142, *P* = .006) for cachexia.

### 3.3. The Genotype and Allele Frequencies of IL-8

The genotype and allele frequencies of the IL-8 gene −251 A/T and +781 C/T polymorphisms for all the studied variations are shown in Tables 3 and 4. Within each study group, the genotype distributions were consistent with those predicted by the Hardy-Weinberg equilibrium.

No significant difference was found in genotype and allele frequencies between the low-third gastric cancer patients and the controls (Table 3). There were significant differences in the genotype frequencies of the IL-8 gene +781 C/T polymorphism between patients with cachexia and without (Table 4) (*P* = .009), and a significantly increased frequency of +781 T allele was noted in patients with cachexia (OR = 2.247, 95% CI: 1.351–3.737, *P* = .002). The +781 TT genotypes were observed to be associated with a significantly increased risk of cachexia (OR = 3.167, 95% CI: 1.265–7.929, *P* = .011), and with odds ratio of 3.033 (95% CI: 1.065–8.639, *P* = .038) for cachexia after adjusting for patients' actual weight and carcinoma stage.

### 3.4. Haplotype Analysis of the IL-8 Gene

Haplotype analyses were performed and the possible four haplotype frequencies are shown in Table 5. Linkage disequilibrium was observed between locus −251 and locus +781 (*D*′ = 0.7). Haplotype analysis revealed major T251C781 haplotype accounted for 45.1% and 56.2% of these four haplotypes in both the cachexia and noncachexia patients, respectively. Haplotype analysis revealed that T^251^T^781^ haplotype (defined by SNPs at positions −251 and +781) was associated with a borderline positive risk of cachexia as compared with the T^251^C^781^ haplotype (OR = 4.92, 95% CI: 1.00–24.28; *P* = .053).

## 4. Discussion

Cancer-related cachexia is typified by poor prognosis and is a significant cause of morbidity and mortality in cancer patients. Various studies have suggested the involvement of certain cytokines in cachexia. On this basis, we examined a cohort of patients with low-third gastric cancer who had cachexia to discuss whether the elevated serum level of IL-8 and its two common −251 A/T and +781 C/T polymorphisms are related to cachexia.

In our study, serum IL-8 level was significantly higher in low-third gastric cancer patients than those in healthy controls, and was further up-regulated in cachectic patients compared with noncachectic patients. In agreement with our results, the correlations of IL-8 concentration with the occurrence of cachexia have been confirmed in other studies [17, 18]. Noticeably, some studies have suggested that the primary tumor and distant metastasis may be associated with the levels of procachectic cytokines in cancer patients. Krzystek-Korpacka et al. [18] demonstrated that both IL-6 and IL-8 were correlated with gastroesophageal cancer T stage. Ueda et al. [32] reported that IL-6 and IL-8 may play an important role in the hematogenous metastasis of colorectal cancer. In view of these, we made logistic regression analysis adjusted for actual weight and tumor stage, and found that IL-8 was the independent predictor of cachexia in low-third gastric cancer patients.

Although the precise mechanisms regulating IL-8 expression remain not well known, several studies have shown that polymorphisms of the IL-8 gene have functional importance. Polymorphisms at position −251 and +781 of the IL-8 gene, especially the A^251^T^781^ haplotype, have been reported to be associated with greater IL-8 production and expression [21, 33, 34]. Tsai et al. [35] have observed significant relationships between the IL-8 +781 T allele and wet age-related macular degeneration (AMD). In our study, a significantly increased frequency of +781 T allele was noted in patients with cachexia, and the +781 TT genotype was significantly associated with the risk of cachexia. Thus it can be conceivable that a higher promoter activity of +781 T allele in IL-8 gene and its TT genotype might increase the expression of IL-8, resulting in a high profuction of IL-8, which may lead to be more susceptible to cachexia.

Previous reports have demonstrated that the −251 A allele within the promoter was signifcantly associated with higher IL-8 levels and is itself a functional variant [33, 36]. In our study, we failed to observe the relation between IL-8 −251 A/T polymorphisms and cachexia in low-third gastric cancer patients, and in contrast, the T^251^T^781^ haplotype of IL-8 gene seemed to be positively associated with cachexia. It just can be conceivable that −251 T allele may participate in up-regulating the production of IL-8 but can not be regarded as an independent predictor of cachexia. In fact, it is still unclear which allele of IL-8 −251 A/T polymorphism is the functional variant that directly affects IL-8 production; the relationship has been controversial [20, 37].

In the current study, the IL-8 +781 T allele frequency among the healthy Chinese Han population in our study was 0.39, which was higher than that of Taiwan Chinese populations (0.28) [35], but was comparable to that found in previous study in healthy American populations (0.35) [32]. To eliminate possible bias, we carefully selected both our low-third gastric cancer and control groups. First, to avoid artifact in population admixture, we selected only Chinese Han people. As age, smoking habits, drinking water status, and helicobacter pylori infection are important risk factor in gastric cancer, the control sample was all matched to the patient group.

In conclusion, our results showed that the serum level of IL-8 and its +781 C/T polymorphism have a significant association with the presence of cachexia in low-third gastric cancer patients. Future studies with larger sample sizes will be needed to validate the genetic effects of the IL-8 polymorphisms on cachexia in gastric cancer. Nevertheless, the results of our study are limited because of the relatively small numbers of cases and controls. Additional studies of the exact putative biologic functions of IL-8 on cachexia are also needed to be elucidated.

## Figures and Tables

**Table 1:** Characteristics of gastric cancer patients in the analysis of cachexia.

	Cachexia	Noncachexia	
	(*n* = 61)	(*n* = 64)	*P*
Sex (M/F)	38/23	45/19	.343^3^
Age (year)	55. 27 ± 12.24^1^	57.26 ± 11.32	.346^2^
Serum albumin (g/L)	28.6 ± 4.7	35.2 ± 5.1	<.001^2^
Actual weight (kg)	64.9 ± 8.2	65.8 ± 12.5	.656^2^
Carcinoma stage			<.001^3^
I	0	14	
II	31	5	
III	17	34	
IV	13	11	

^1^Mean ± SD (all such values).

^2^2-sample*t* test.

^3^Pearson chi-square test.

**Table 2:** Characteristics of the cancer patients and controls.

	Cancer	Contr.	
	(*n* = 125)	(*n* = 140)	*P*
Sex (M/F)	83/42	99/41	.450^3^
Age (year)	56.18 ± 11.78^1^	54.24 ± 12.03	.185^2^
Smoking status			0.563^3^
never	41 (32.8%)	54 (38.6%)	
Ex-	24 (19.2%)	22 (15.7%)	
current	60 (48.0%)	64 (45.7%)	
Helicobacter pylori infection	74 (59.2%)	76 (54.3%)	.420^3^
Poor drinking water	18 (14.4%)	13 (9.3%)	.196^3^

^1^Mean ± SD (all such values).

^2^2-sample *t* test.

^3^Pearson chi-square test.

**Table 3:** The genotype and allele frequencies of IL-8 polymorphism in cancer patients and controls.

Polymorphism	Cancer *n* = 125 (%)	Contr. *n* = 140 (%)	*χ* ^2^	*P*
IL-8 −251 A/T			1.659	.436
AA	20 (16.0)	23 (16.4)		
AT	72 (57.6)	70 (50.0)		
TT	33 (26.4)	47 (35.6)		
A allele	112 (44.8)	116 (41.4)	0.612	.434
T allele	138 (55.2)	164 (58.6)		
IL-8 +781 C/T			1.356	.508
CC	40 (32.0)	53 (37.9)		
CT	58 (46.4)	63 (45.0)		
TT	27 (21.6)	24 (17.1)		
C allele	138 (55.2)	169 (60.4)		
T allele	112 (44.8)	111 (39.6)	1.441	.230

**Table 4:** The genotype and allele frequencies of IL-8 polymorphism in patients with gastric cancer.

Polymorphism	Cachexia *n* = 61 (%)	Noncachexia *n* = 64 (%)	*χ* ^2^	*P*
IL-8 −251 A/T			1.224	.542
AA	12 (19.7)	8 (12.5)		
AT	34 (55.7)	38 (59.4)		
TT	15 (24.6)	18 (28.1)		
A allele	58 (47.5)	54 (42.2)	0.724	.395
T allele	64 (52.5)	74 (57.8)		
IL-8 +781 C/T			9.315	.009
CC	13 (21.3)	27 (42.2)		
CT	29 (47.5)	29 (45.3)		
TT	19 (31.2)	8 (12.5)		
C allele	55 (45.1)	83 (64.8)		
T allele	67 (54.9)	45 (35.2)	9.864	.002

**Table 5:** IL-8 gene haplotype distribution in the patients with cachexia and with noncachexia.

Haplotype	Cachexia 2*n* = 122 (%)	Noncachexia 2*n* = 128 (%)	Odds ratio (95% confidence interval)	*P*
T^251^C^781^	55 (45.1)	72 (56.2)	1.00	
A^251^T^781^	58 (47.5)	43 (33.6)	1.48 (0.84 − 2.61)	.17
A^251^C^781^	0	11 (8.6)	0.00 (-Inf - Inf)	1
T^251^T^781^	9 (7.4)	2 (1.6)	4.92 (1.00 − 24.28)	.053

## References

[1] Tan BHL, Deans DAC, Skipworth RJE, Ross JA, Fearon KCH (2008). Biomarkers for cancer cachexia: is there also a genetic component to cachexia?. *Supportive Care in Cancer*.

[2] Luft FC (2007). Cachexia has only one meaning. *Journal of Molecular Medicine*.

[3] Zhang D (2009). Probing cancer cachexia-anorexia: recent results with knockout, transgene and polymorphisms. *Curr Opin Clin Nutr Metab Care*.

[4] Tisdale MJ (2005). Molecular pathways leading to cancer cachexia. *Physiology*.

[5] Kotler DP (2000). Cachexia. *Annals of Internal Medicine*.

[6] Argils JM, Meijsing H, Pallars-Trujillo J, Guirao X, Lpez-Soriano FJ (2001). Cancer cachexia: a therapeutic approach. *Medicinal Research Reviews*.

[7] Muscaritoli M, Bossola M, Aversa Z, Bellantone R, Rossi Fanelli F (2006). Prevention and treatment of cancer cachexia: new insights into an old problem. *European Journal of Cancer*.

[8] Argiles JM, Busquets S, Garcia-Martonez C, Lopez-Soriano FJ (2005). Mediators involved in the cancer anorexia-cachexia syndrome: past, present, and future. *Nutrition*.

[9] McNamara MJ, Alexander HR, Norton JA (1992). Cytokines and their role in the pathophysiology of cancer cachexia. *Journal of Parenteral and Enteral Nutrition*.

[10] Tisdale MJ (2000). Biomedicine: protein loss in cancer cachexia. *Science*.

[11] Tisdale MJ (2000). Metabolic abnormalities in cachexia and anorexia. *Nutrition*.

[12] Tisdale MJ (1998). New cachexic factors. *Current Opinion in Clinical Nutrition and Metabolic Care*.

[13] Tisdale MJ (1997). Isolation of a novel cancer cachectic factor. *Proceedings of the Nutrition Society*.

[14] Matsushima K, Morishita K, Yoshimura T (1988). Molecular cloning of a human monocyte-derived neutrophil chemotactic factor (MDNCF) and the induction of MDNCF mRNA by interleukin 1 and tumor necrosis factor. *Journal of Experimental Medicine*.

[15] Matsushima K, Baldwin ET, Mukaida N (1992). Interleukin-8 and MCAF: novel leukocyte recruitment and activating cytokines. *Chemical Immunology*.

[16] Maeda S, Ogura K, Yoshida H (1998). Major virulence factors, VacA and CagA, are commonly positive in Helicobacter pylori isolates in Japan. *Gut*.

[17] Pfitzenmaier J, Vessella R, Higano CS, Noteboom JL, Wallace D, Corey E (2003). Elevation of cytokine levels in cachectic patients with prostate carcinoma. *Cancer*.

[18] Krzystek-Korpacka M, Matusiewicz M, Diakowska D (2007). Impact of weight loss on circulating IL-1, IL-6, IL-8, TNF-*α*, VEGF-A, VEGF-C and midkine in gastroesophageal cancer patients. *Clinical Biochemistry*.

[19] Ross OA, O'Neill C, Rea IM (2004). Functional promoter region polymorphism of the proinflammatory chemokine IL-8 gene associates with Parkinson's disease in the Irish. *Human Immunology*.

[20] Ohyauchi M, Imatani A, Yonechi M (2005). The polymorphism interleukin 8 −251A/T influences the susceptibility of Helicobacter pylori related gastric diseases in the Japanese population. *Gut*.

[21] Hacking D, Knight JC, Rockett K (2004). Increased in vivo transcription of an IL-8 haplotype associated with respiratory syncytial virus disease-susceptibility. *Genes and Immunity*.

[22] Vogiatzi K, Apostolakis S, Voudris V, Thomopoulou S, Kochiadakis GE, Spandidos DA (2008). Interleukin 8 and susceptibility to coronary artery disease: a population genetics perspective. *Journal of Clinical Immunology*.

[23] Vairaktaris E, Yapijakis C, Serefoglou Z (2007). The interleukin-8 (−251A/T) polymorphism is associated with increased risk for oral squamous cell carcinoma. *European Journal of Surgical Oncology*.

[24] Snoussi K, Mahfoudh W, Bouaouina N, Ahmed SB, Helal AN, Chouchane L (2006). Genetic variation in IL-8 associated with increased risk and poor prognosis of breast carcinoma. *Human Immunology*.

[25] Wei Y-S, Lan Y, Tang R-G (2007). Single nucleotide polymorphism and haplotype association of the interleukin-8 gene with nasopharyngeal carcinoma. *Clinical Immunology*.

[26] Zhang D, Zhou Y, Wu L (2008). Association of IL-6 gene polymorphisms with cachexia susceptibility and survival time of patients with pancreatic cancer. *Annals of Clinical and Laboratory Science*.

[27] Deans C, Rose-Zerilli M, Wigmore S (2007). Host cytokine genotype is related to adverse prognosis and systemic inflammation in gastro-oesophageal cancer. *Annals of Surgical Oncology*.

[28] Zhang D, Zheng H, Zhou Y, Tang X, Yu B, Li J (2007). Association of IL-1 beta gene polymorphism with cachexia from locally advanced gastric cancer. *BMC Cancer*.

[29] Sobin LH, Wittekind Ch (2002). *TNM Classification of Malignant Tumors*.

[30] Fearon KC, Voss AC, Hustead DS (2006). Definition of cancer cachexia: effect of weight loss, reduced food intake, and systemic inflammation on functional status and prognosis. *American Journal of Clinical Nutrition*.

[31] Heinzmann A, Ahlert I, Kurz T, Berner R, Deichmann KA (2004). Association study suggests opposite effects of polymorphisms within IL8 on bronchial asthma and respiratory syncytial virus bronchiolitis. *Journal of Allergy and Clinical Immunology*.

[32] Ueda T, Shimada E, Urakawa T (1994). Serum levels of cytokines in patients with colorectal cancer: possible involvement of interleukin-6 and interleukin-8 in hematogenous metastasis. *Journal of Gastroenterology*.

[33] Taguchi A, Ohmiya N, Shirai K (2005). Interleukin-8 promoter polymorphism increases the risk of atrophic gastritis and gastric cancer in Japan. *Cancer Epidemiology Biomarkers and Prevention*.

[34] Itoh Y, Joh T, Tanida S (2005). IL-8 promotes cell proliferation and migration through metalloproteinase-cleavage proHB-EGF in human colon carcinoma cells. *Cytokine*.

[35] Tsai Y-Y, Lin J-M, Wan L (2008). Interleukin gene polymorphisms in age-related macular degeneration. *Investigative Ophthalmology and Visual Science*.

[36] Hull J, Thomson A, Kwiatkowski D (2000). Association of respiratory syncytial virus bronchiolitis with the interleukin 8 gene region in UK families. *Thorax*.

[37] Lee W-P, Tai D-I, Lan K-H (2005). The -251T allele of the interleukin-8 promoter is associated with increased risk of gastric carcinoma featuring diffuse-type histopathology in Chinese population. *Clinical Cancer Research*.

